# Shenling Baizhu San ameliorates non-alcoholic fatty liver disease in mice by modulating gut microbiota and metabolites

**DOI:** 10.3389/fphar.2024.1343755

**Published:** 2024-04-24

**Authors:** Dongliang Chen, Yuanfei Wang, Jianmei Yang, Wanyi Ou, Guiru Lin, Ze Zeng, Xiaomin Lu, Zumin Chen, Lili Zou, Yaling Tian, Aiping Wu, Shelley E. Keating, Qinhe Yang, Chenli Lin, Yinji Liang

**Affiliations:** ^1^ School of Nursing, Jinan University, Guangzhou, Guangdong Province, China; ^2^ School of Medicine, Jinan University, Guangzhou, Guangdong Province, China; ^3^ School of Human Movement and Nutrition Sciences, The University of Queensland, Brisbane, QLD, Australia; ^4^ School of Chinese Medicine, Jinan University, Guangzhou, Guangdong Province, China; ^5^ Health Science Center, Jinan University, Guangzhou, Guangdong Province, China

**Keywords:** non-alcoholic fatty liver disease, gut microbiota, metabolites, traditional Chinese medicine, metabolic-associated steatotic liver disease

## Abstract

**Purpose:** The prevalence of non-alcoholic fatty liver disease (NAFLD) and its related mortality is increasing at an unprecedented rate. Traditional Chinese medicine (TCM) has been shown to offer potential for early prevention and treatment of NAFLD. The new mechanism of “Shenling Baizhu San” (SLBZS) is examined in this study for the prevention and treatment of NAFLD at the preclinical level.

**Methods:** Male C57BL/6J mice were randomly divided into three groups: normal diet (ND), western diet + CCl_4_ injection (WDC), and SLBZS intervention (WDC + SLBZS). Body weights, energy intake, liver enzymes, pro-inflammatory factors, and steatosis were recorded in detail. Meanwhile, TPH1, 5-HT, HTR2A, and HTR2B were tested using qRT-PCR or ELISA. Dynamic changes in the gut microbiota and metabolites were further detected through the 16S rRNA gene and untargeted metabolomics.

**Results:** SLBZS intervention for 6 weeks could reduce the serum and liver lipid profiles, glucose, and pro-inflammatory factors while improving insulin resistance and liver function indexes in the mice, thus alleviating NAFLD in mice. More importantly, significant changes were found in the intestinal TPH-1, 5-HT, liver 5-HT, and related receptors HTR2A and HTR2B. The 16S rRNA gene analysis suggested that SLBZS was able to modulate the disturbance of gut microbiota, remarkably increasing the relative abundance of probiotics (*Bifidobacterium* and *Parvibacter*) and inhibiting the growth of pro-inflammatory bacteria (*Erysipelatoclostridium* and *Lachnoclostridium*) in mice with NAFLD. Combined with metabolomics in positive- and negative-ion-mode analyses, approximately 50 common differential metabolites were selected via non-targeted metabolomics detection, which indicated that the targeting effect of SLBZS included lipid metabolites, bile acids (BAs), amino acids (AAs), and tryptophan metabolites. In particular, the lipid metabolites 15-OxEDE, vitamin D3, desoxycortone, and oleoyl ethanol amide were restored by SLBZS.

**Conclusion:** Integrating the above results of multiple omics suggests that SLBZS ameliorates NAFLD via specific gut microbiota, gut-derived 5-HT, and related metabolites to decrease fat accumulation in the liver and inflammatory responses.

## 1 Introduction

Non-alcoholic fatty liver disease (NAFLD) is a global health problem that poses substantial healthcare burden for many countries. NAFLD has an estimated prevalence of up to 30% worldwide (Younossi et al., 2023), with China showing rapid growth and prevalence of about 29%, notably among young individuals (Zhou et al., 2020). According to the degree of inflammation and fibrosis in NAFLD, the spectrum of disease states can extend from simple liver steatosis, non-alcoholic steatohepatitis, liver fibrosis, and cirrhosis to hepatocellular carcinoma (Cobbina and Akhlaghi, 2017). Moreover, NAFLD is an important pathogenic risk factor for type 2 diabetes, cardiovascular disease, and extrahepatic carcinoma (Powell et al., 2021). Thus, early lifestyle interventions involving diet and regular exercise are the cornerstone of NAFLD prevention and management. Lifestyle therapies can help reduce liver fat, improve liver health and metabolic comorbidities, and decrease NAFLD-related morbidity (Rinella et al., 2023). Although lifestyle therapy is the primary approach to management, several pharmacological agents to treat NAFLD have entered clinical trials, such as the PPARα/δ agonist elafibranor, FXR agonist obeticholic acid, and CCR2/CCR5 inhibitor cenicriviroc. However, there are no approved agents available for use (Patel et al., 2020; Ratziu et al., 2020; Huang et al., 2021). There is a growing body of evidence indicating that traditional Chinese medicine (TCM) can prevent and treat early-stage NAFLD (Dang et al., 2020; Jiang et al., 2022); TCM may therefore complement lifestyle approaches to preventing and treating NAFLD.

The pathogenesis of NAFLD is multifactorial, and a clear understanding of the mechanisms involved is impeded by the lack of sufficient non-invasive biomarkers. The currently accepted hypothesis is a “parallel, multiple-hit model” involving dietary factors, inflammatory activation, insulin resistance, adipose tissue dysfunction, and gut microbiota dysfunction as participants in the progression of NAFLD (Buzzetti et al., 2016; Loomba et al., 2021). Gut microbiota are intricately connected with many chronic diseases (Wang et al., 2021). In the state of eubiosis, the host and gut microbiota mutually benefit each other under healthy conditions. Microbiome dysbiosis could be caused by an abnormal ratio of commensal to pathogenic bacterial species and has been shown to have direct associations with inflammatory and metabolic disorders (Pinart et al., 2021; Hosomi et al., 2022), NAFLD (Wang W. et al., 2020; Amini-Salehi et al., 2023), and diabetes (Zhang et al., 2020; Ojo et al., 2021). Preclinical and clinical evidence suggest that modulation of the gut microbiome might represent a new therapeutic target for people with NAFLD. There is increasing interest in the treatment and management of NAFLD using microbiome-targeted therapies (MTTs) (Sharpton et al., 2019; Wang W. et al., 2020; Amini-Salehi et al., 2023). While the mechanism of the gut microbiota in treating NAFLD is not fully understood, metabolites derived from the gut microbiota may play key roles in its pathogenesis (Vallianou et al., 2021).

TCM is underpinned by the theory of holism and differentiation treatment. Owing to its multi-ingredient and multitarget regulation, TCM has unique advantages for targeting the complex pathogenesis of NAFLD (Dai et al., 2021). In recent years, studies have shown that TCM approaches could prevent NAFLD by modulating the structures and functions of the gut microbiota and metabolites. For example, *Penthorum chinense Pursh* has been shown to attenuate high-fat diet-induced NAFLD by regulating the gut microbiota and bile acid (BA) metabolism in mice (Li X. et al., 2022). Lingguizhugan decoction has been shown to improve insulin resistance (IR), hepatic steatosis, and non-alcoholic steatohepatitis (NASH) by modulating the gut microbiota and correlated metabolites (Ning et al., 2022; Zhu et al., 2023), such as BAs, amino acids (AAs), and short-chain fatty acids (SCFAs). Therefore, gut microbiota and correlated metabolites have emerged as novel therapeutic strategies for potential TCM interventions in NAFLD.

Shenling Baizhu San (SLBZS) is a TCM that was first proposed in “Tai Ping Hui Min He Ji Ju” in the Song Dynasty; it is known to invigorate spleen function and has been widely used in treating gastrointestinal and liver diseases (Deng et al., 2018; Chao et al., 2020). Previous studies have demonstrated that SLBZS improved colitis by modulating gut microbiota dysbiosis (Jiao et al., 2022; Lv et al., 2022), which is also an important risk factor for NAFLD (Aron-Wisnewsky et al., 2020; Xue et al., 2022). SLBZS has also been shown to improve liver inflammation, decrease liver lipid accumulation, and reduce liver steatosis (Pan et al., 2019; Pan et al., 2021), making it potentially beneficial for NAFLD prevention and management. However, the effects of SLBZS on NAFLD and its potential mechanism from the perspective of the gut microbiota and related metabolites are unknown.

A new mouse model of NAFLD, which is particularly similar to human genetic and metabolic changes, is used in this study to evaluate the effects of SLBZS on NAFLD (Tsuchida et al., 2018). Moreover, 16S rRNA gene sequencing and UHPLC-MS/MS technologies were combined to profile the alterations in gut microbiota and metabolites in fecal samples. These results may explain the potential mechanisms of SLBZS in treating NAFLD and provide the candidate microbiota and metabolites for alleviating NAFLD.

## 2 Materials and methods

### 2.1 Animals and drugs

Male C57BL/6J mice (six to eight weeks) were purchased from the Laboratory Animal Technology Co., Ltd. of Zhejiang Wei Tong Li Hua (Beijing, China). All animals were housed at a temperature of 23 ± 2°C and humidity of 55% ± 5% under a 12-h light–dark cycle with free access to food and water at the Animal Center of Jinan University (Zou et al., 2023). All procedures were conducted in accordance with the Ethics Committee of Jinan University, China (No. IACUC-20211029-11). All Chinese medical herbs were purchased from Guangdong Hospital of Traditional Chinese Medicine (Guangzhou, China). SLBZS is composed of ten Chinese medicinal herbs, as listed in Supplementary Table S1.

### 2.2 Compounds in SLZBS by UHPLC-MS/MS

The compounds in SLZBS were analyzed using the Vanquish™ ultrahigh-performance liquid chromatography (UHPLC) system (Thermo Scientific, United States). The chromatographic column used was the Accucore column (C18, 150 mm × 2.1 mm, 1.8 μm), with a column temperature of 35°C, flow rate of 0.3 mL/min, and total time of 30 min. The mobile phase comprised 0.1% aqueous formic acid solution (solvent A) and methanol (solvent B). The gradient elution conditions are shown in Supplementary Table S2. The UHPLC system was combined with a benchtop Q Exactive hybrid quadrupole-Orbitrap mass spectrometer (Thermo Scientific). The mass spectrometry information of SLBZS was collected by the positive- and negative-ion modes of the electrospray ionization source (ESI) (Supplementary Figure S1; Supplementary Table S3).

### 2.3 Experimental design and drug administration

The mice were randomly divided into three groups (n = 6 for each group) as follows: ND (normal diet, no CCl_4_ injection), WDC (Western diet, CCl_4_ injection), and WDC + SLBZS (SLBZS, Western diet, CCl_4_ injection) (Tsuchida et al., 2018). The mice were fed a normal chow diet (ND, 5C02, Lab diet) and daily drinking water or WD containing 17.3% protein, 48.5% carbohydrates, and 21.2% fat by weight (TP.120528A, Trophic Animal Feed High-tech Co., Ltd., China) along with a high-sugar solution containing 23.1 g/L d-fructose (F108334, Aladdin) and 18.9 g/L d-glucose (G116306, Aladdin). A low dose of CCl_4_ (10006464, Sinopharm Chemical Reagent Co., Ltd., China) at 0.2 µL/g of body weight dissolved in corn oil (10% CCL_4_/corn oil) or its control vehicle corn oil was injected intraperitoneally once per week. The WDC + SLBZS group was orally administered SLBZS at 21.8 g/kg every day for 6 weeks (Reagan-Shaw et al., 2008), while the ND and WDC groups were given equal volumes of distilled water. The body weights and food intake of the mice were recorded once a week. All mice were euthanized at 6 weeks, and their liver, serum samples, feces, and small intestine were collected.

### 2.4 Liver histology

The liver and small intestine tissue sections were fixed in 4% paraformaldehyde, embedded in paraffin wax, cut into 5-μm sections, and mounted on glass slides. The sections were stained with hematoxylin and eosin (H&E) for assessment of liver histology (Zhang et al., 2021). The NAFLD activity score was evaluated by two pathologists according to the NASH CRN scoring system, and all slides were blindly scored (Kleiner et al., 2005). The frozen liver tissue used for Oil Red O (ORO) staining was embedded in an optimal cutting temperature compound and sectioned to 5 μm thickness (Zhang et al., 2021). Images were obtained under a microscope (Leica, Germany), and semi-quantitative analysis of the ORO staining areas was achieved using ImageJ (Li Q. et al., 2022).

### 2.5 Immunohistochemistry

The small intestine sections in paraffin were baked, deparaffinized, hydrated, antigenically repaired (sodium citrate pH 6.0, microwave heating), endogenous catalase inactivated, sealed with goat serum and slides incubated with primary antibody E-cadherin (GB12083, Servicebio, China) at 4°C overnight, and incubated with secondary antibody HRP-labeled goat anti-rabbit IgG (GB23303, Servicebio, China) at room temperature for 50 min; then, DAB chromatography (G1212, Servicebio, China) was used to detect the expression of E-cadherin in the small intestine samples after application of biotin amphiphile to amplify the signals.

### 2.6 Serum and liver biochemical parameters

Serum alanine aminotransferase (ALT), aspartate aminotransferase (AST), hepatic triglyceride (TG), and total cholesterol (TC) levels were determined by using commercial assay kits (Nanjing Jian Cheng Bioengineering Institute, China) according to manufacturer’s protocols. An intraperitoneal glucose tolerance test (IPGTT) was performed in the mice after 12 h of fasting with free drinking. Glucose (2 mg/g body weight) in normal water was administered to the mice via intraperitoneal injection. The blood glucose (BG) levels were measured from the tail blood at 0, 30, 60, 90, and 120 min after glucose administration (Contour TS, Bayer) (Welch et al., 2021). The concentrations of 5-HT in the liver and small intestine were analyzed by using an enzyme-linked immunosorbent assay (ELISA) kit according to manufacturer’s protocols (Zou et al., 2023).

### 2.7 Real-time reverse transcriptase polymerase chain reaction (RT-PCR) analysis

RNA was extracted from 20 mg of the liver and small intestine tissues and purified (RC101, VAZYME, America). Approximately 1 µg of the total RNA was reverse-transcribed using a complementary DNA conversion kit (R223-01, VAZYME, United States), and the gene expression levels were determined by quantitative PCR (Q712, VAZYME, United States) using the CFX Connect Real-Time PCR Detection System (BIO-RAD) (Zou et al., 2023). The first step of the qRT-PCR protocol was 95°C for 30 s, followed by 40 cycles for 3 s at 95°C, 10 s at 60°C, 15 s at 95°C, 60 s at 60°C, and 15 s at 95°C each. The relative expressions of the target genes were normalized with respect to GAPDH expression as the internal control. The primer sequences are presented in Table 1.

**TABLE 1:** Primer sets used in the present study.

Gene	Primer sequences (5′–3′)
**GAPDH**	Forward: TCA​ACA​GCA​ACT​CCC​ACT​CTT​CCA
	Reverse: TTG​TCA​TTG​AGA​GCA​ATG​CCA​GCC
**IL-6**	Forward: GTG​ACA​ACC​ACG​GCC​TTC​CCT​ACT
	Reverse: GGTAGCTATGGT ACTCCA
**TNF-α**	Forward: GCG​ACG​TGG​AAC​TGG​CAG​AAG
	Reverse: GGT​ACA​ACC​CAT​CGG​CTG​GCA
**MCP-1**	Forward: TCTG GGCCTGCTGTTCACA
	Reverse: GGA​TCA​TCT​TGC​TGG​TGA​ATG​A
**IL-1β**	Forward: GAA​ATG​CCA​CCT​TTT​GAC​AGT​G
	Reverse: TGGATGC TCTCATCAGGACAG
**IL-10**	Forward: ATA​ACT​GCA​CCC​ACT​TCC​CA
	Reverse: GGG​CAT​CAC​TTC​TAC​CAG​GT
**IL-18**	Forward: ACA​ACT​TTG​GCC​GAC​TTC​AC
	Reverse: ATC​AGT​CTG​GTC​TGG​GGT​TC
**HTR2A**	Forward: TAA​TGC​AAT​TAG​GTG​ACG​ACT​CG
	Reverse: GAG​GCT​TCG​GAA​GTG​TTA​GCA
**HTR2B**	Forward: ACC​TGA​TCC​TGA​CTA​ACC​GT
	Reverse: TGG​GTA​TAT​CAC​CGC​GAG​TAT
**TPH-1**	Forward: ACC​ATG​ATT​GAA​GAC​AAC​AAG​GAG
	Reverse: TCA​ACT​GTT​CTC​GGC​TGA​TG

### 2.8 Gut microbiota 16S rRNA gene analysis

Mice stools (n = 6) were collected at 6 weeks and stored at −80°C. DNA from the microbial community in the mice stools was extracted using the HiPure Stool DNA Kit. The quality and concentration of the extracted DNA samples were evaluated using NanoDrop microvolume spectrophotometry (NanoDrop 2000; Thermo Fisher Scientific, United States). The V3–V4 variable region of the 16S rRNA gene was amplified by PCR. Amplicons were extracted from 2% agarose gels and purified with AMPure XP Beads (Beckman Agencourt, United States), followed by quantification on the ABI StepOnePlus Real-Time PCR System (Life Technologies, Foster City, United States). The purified samples were sequenced and analyzed based on the PE250 mode pooling test of NovaSeq 6000. The sequencing service was provided by Genedenovo Inc. (Guangzhou, China) (Magoč and Salzberg, 2011; Bokulich et al., 2013; Edgar, 2013).

### 2.9 Fecal untargeted metabolomics based on UHPLC-MS/MS analysis

Metabolites were extracted from the mice stools using lipid nitrogen and methanol (Want et al., 2013). To obtain reliable and high-quality data, QS samples were used as quality controls. UHPLC-MS/MS analyses were performed using the Vanquish UHPLC system (Thermo Fisher, Germany) coupled with an Orbitrap Q Exactive™ HF-X mass spectrometer (Thermo Fisher, Germany). Chromatographic separation was then performed on a Hypesil Gold column (100 × 2.1 mm, 1.9 μm).

### 2.10 Statistical and bioinformatics analyses

Statistical analyses were performed using the statistical software R (R version R-3.4.3), Python (Python 2.7.6 version), and CentOS (CentOS release 6.6), with graphing using GraphPad Prism 8.0 (GraphPad Software, San Diego, CA, United States). The Wilcoxon rank-sum test was used for comparisons between the control and NAFLD group, and NAFLD and SLBZS groups, while the Kruskal–Wallis rank-sum test and one-way analysis of variance (ANOVA) followed by Tukey’s multiple comparison test were conducted to assess the differences among the three groups. The data were expressed in terms of mean ± standard deviation (SD). A *p*-value < 0.05 was deemed to be statistically significant. For further details regarding the materials used, the readers are referred to Supplementary Table S6.

## 3 Results

### 3.1 SLBZS improves obesity and blood glucose level in NAFLD mice

**FIGURE 1:**
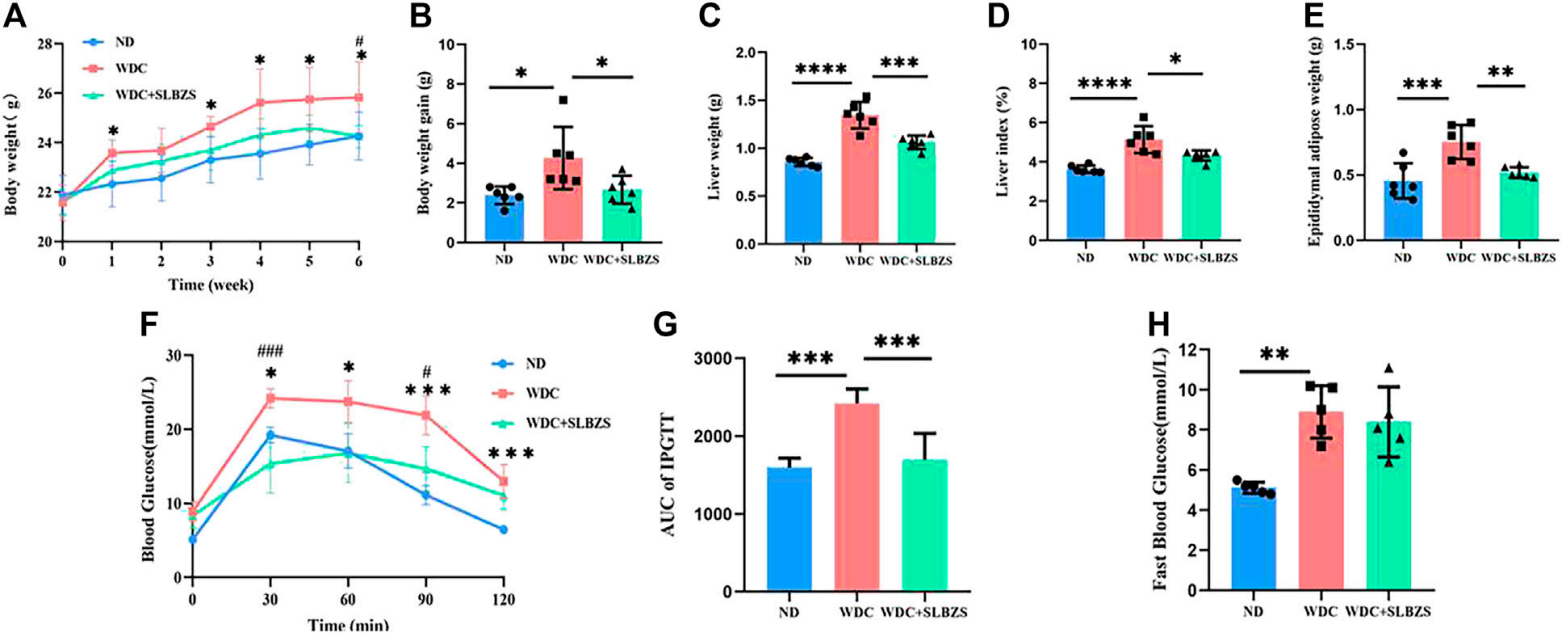
SLBZS ameliorated obesity and blood glucose in NAFLD mice. **(A, B)** Weekly body weight and body weight gain. **(C)** Liver weight. **(D)** Liver index (Liver weight/Body weight). **(E)** Epididymal adipose weight. **(F)** IPGTT. **(G)**The area under the curve of IPGTT. **(H)** Fast Blood Glucose. Data are presented as the mean ± SD (n=5-6), *P < 0.05 or **P< 0.01 or ***P < 0.001 or****P < 0.0001 in NALFD‐vs.‐Control group, #P< 0.05 or ##P < 0.01 or ###P< 0.001 in SLBZS‐vs.‐NAFLD group, P value obtained by one‐way ANOVA with Tukey's post hoc tests.

A Western diet along with a low-dose injection of CCl_4_ was used to establish the proposed NAFLD mouse model. Results of the 6-week preventive experiment showed significant body weight gain in the NAFLD group than in the control group. However, the body weight gain in the SLBZS group was similar to that of the control group at the end of the sixth week of treatment (Figures 1A, B). Additionally, there were no differences in the food intake and energy intake between the NAFLD and SLBZS groups (Supplementary Figures S2A, B). Compared with the NAFLD group, the SLBZS group showed significantly reduced liver weight, liver-to-body-weight ratio, and epididymal adipose weight (Figures 1C–E). Moreover, IPGTT was performed to evaluate whether glucose tolerance was altered in the NAFLD and SLBZS groups, and it was found that SLBZS significantly improved insulin resistance (Figures 1F–H). Thus, these data suggested that SLBZS improves obesity and blood glucose level in the NAFLD model.

### 3.2 SLBZS ameliorates liver injury and inflammation in NAFLD mice

**FIGURE 2:**
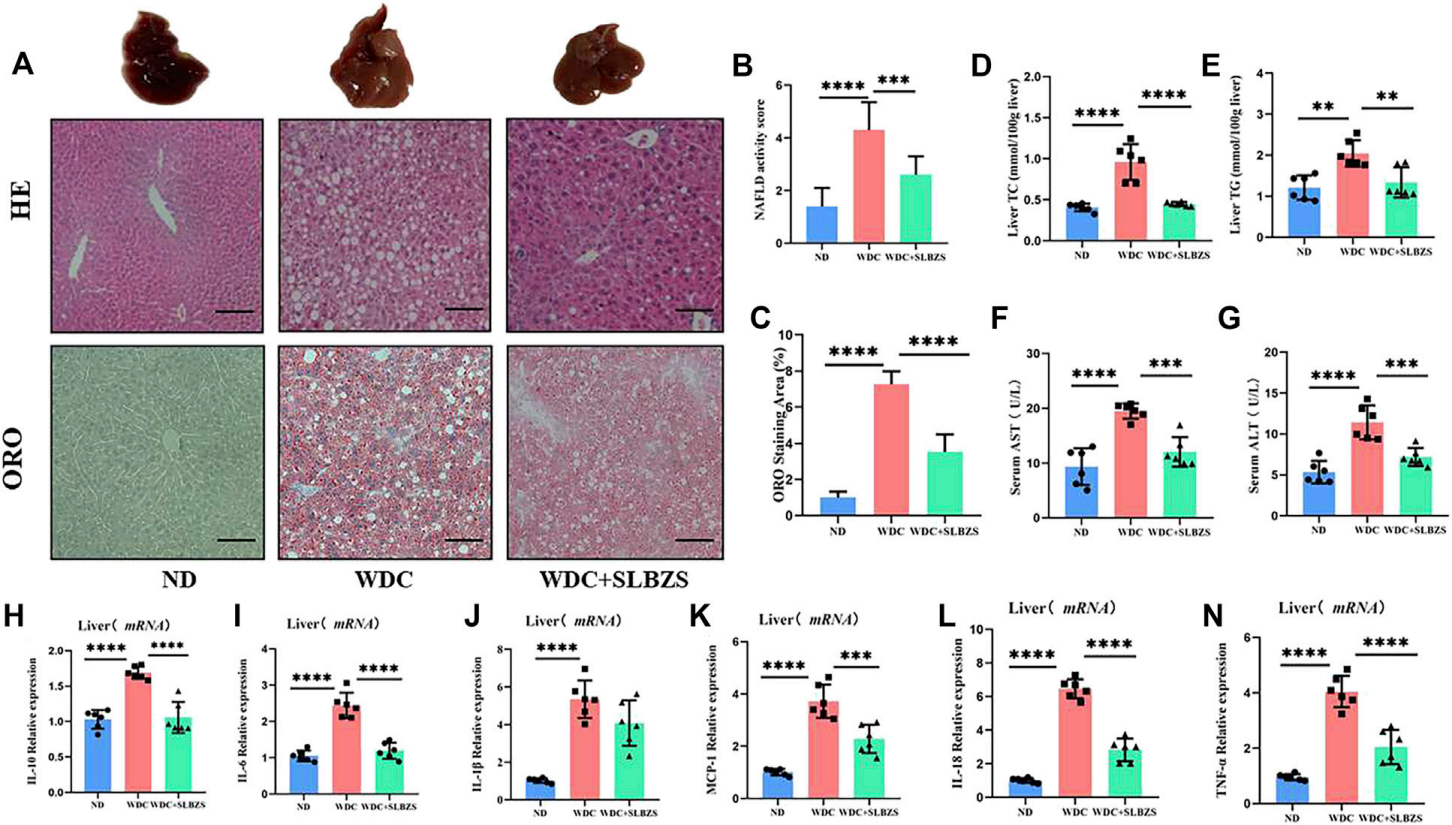
Liver pathology and altered inflammatory factors gene expression in NAFLD mice. **(A)** Representative images of gross morphology, liver tissues stained with H&E and oil red O in ND, WDC and WDC+SLBZS group (scale bar=100um, original magnification ×200). **(B)** NAFLD activity score. **(C)** Semi‐quantitative analysis of oil red O staining area. **(D)** Liver Total Cholesterol(n=6). **(E)** Liver Triglycerides (n=6). **(F)** Serum AST (n=6). **(G)** Serum ALT (n=6). **(H‐N)** Relative mRNA expression of the liver inflammatory factorsIL‐10, IL‐6, IL‐1β, MCP‐1, IL‐18, TNF‐α. Data are presented as the mean ± SD (n=6), *P < 0.05, **P< 0.01, ***P < 0.001, ****P < 0.0001. P value obtained by one-way ANOVA with Tukey's post hoc tests.

Representative pictures of the gross morphology as well as H&E and ORO staining from mice in each group are shown (Figure 2A). The liver from NAFLD mice showed typical pathological features of NAFLD with hepatic steatosis and lipid droplets. After administration of SLBZS, the lesions including hepatocyte steatosis were markedly improved; the NAFLD activity score (NAS) was significantly higher in the NAFLD than in the control group, both of which were significantly lowered after SLBZS intervention (Figure 2B). Additionally, the lipid droplets in the SLBZS group became smaller, with reduced percentage of lipid droplets (Figure 2C). At the end of 6 weeks of treatment, the levels of hepatic TC and TG in the NAFLD group were notably higher than those of the control group. After treatment with SLBZS, the hepatic TC and TG levels were downregulated significantly (Figures 2D, E). Correspondingly, the NAFLD group displayed a significant increase in serum AST and ALT values, which decreased significantly by SLBZS (Figures 2F, G). To validate the anti-inflammatory effects of SLZBS, the mRNA expressions of the inflammatory factors in the liver were tested. It was found that the expressions of IL-10, IL-6, IL-1β, MCP-1, IL-18, and TNF-α of the NAFLD group increased markedly, while these were inhibited after SLBZS administration, except for IL-1β (Figures 2H–N). To summarize, SLBZS was expected to play a protective role against NAFLD by reducing hepatocyte steatosis, lipid droplets, and expression of inflammatory factors.

### 3.3 SLBZS alleviates NAFLD by regulating 5-HT and related receptors

**FIGURE 3:**
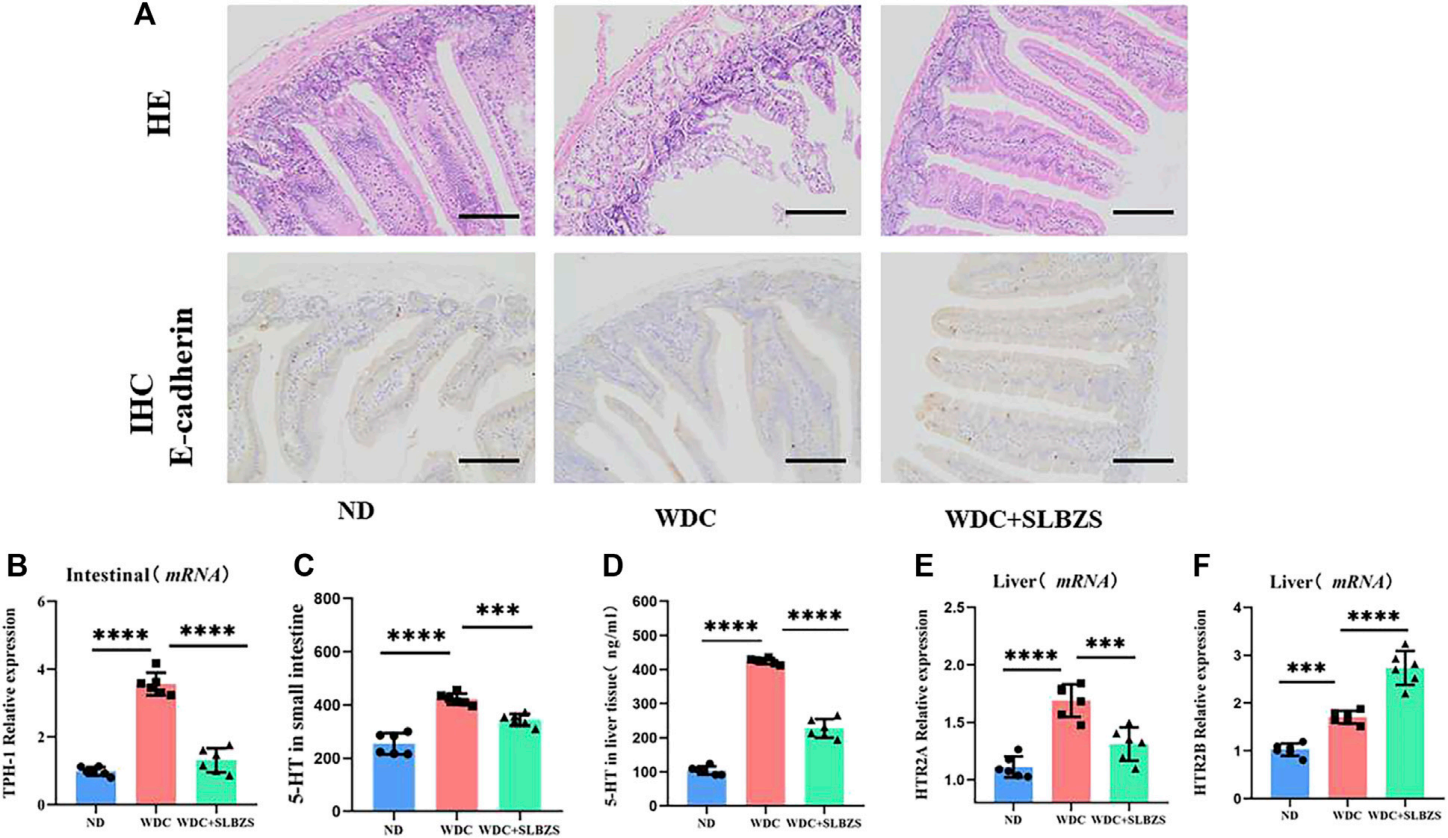
Altered intestinal barrier integrity and serotonin-related signaling pathways in NAFLD mice. **(A)** Representative images of small intestine stained with H&E and IHC of expression of E‐cadherin in ND, WDC and WDC+SLBZS group (scale bar=100um, original magnification ×200). **(B)** Relative mRNA expression of the TPH‐1 in small intestine. **(C)** The serotonin level in small intestine. **(D)** The serotonin level in liver tissue. **(E)** Relative mRNA expression of the HTR2A in liver tissue. **(F)** Relative mRNA expression of the HTR2B in liver tissue. Data are presented as the mean ± SD(n=6), *P < 0.05, **P< 0.01, ***P < 0.001, ****P < 0.0001. P value obtained by one-way ANOVA with Tukey's post hoc tests.

Peripheral 5-HT is produced predominantly by the enterochromaffin (EC) cells in the intestine via tryptophan hydroxylase 1 (TPH-1) (Jin et al., 2021). The overexpression of the 5-HT receptor has been considered a risk factor for hepatic steatosis (Choi et al., 2019). Thus, the expressions of peripheral 5-HT and its related receptors were detected in the small intestine and liver in the experimental mice. With respect to intestine barrier integrity, it was observed that compared with the control group, mice in the NAFLD group showed necrosis and shedding of the mucosal epithelial cells, glandular atrophy, and lumen expansion, which tended to become normal after SLBZS intervention. To further investigate the intestinal tightness, immunohistochemistry was used to color the E-cadherin protein; it was found that the expression level of E-cadherin protein increased after intervention with SLBZS (Figure 3A). Compared to the control group, the mRNA expression of TPH-1 in the small intestine was significantly higher in the NAFLD group, which was decreased by SLBZS (Figure 3B). Moreover, the expressions of 5-HT in the liver and small intestine were elevated significantly in the NAFLD group but declined in the SLBZS group (Figures 3C, D). The results also suggested that the expressions of HTR2A and HTR2B in the liver increased in the NAFLD than in the control group. After administration of SLBZS, the expression of HTR2A was downregulated obviously in contrast to the NAFLD group, except for HTR2B (Figures 3E, F). Thus, SLZBS may mitigate hepatic steatosis by inhibiting the expressions of HTR2A in the liver and TPH-1 in the small intestine as well as by reducing the levels of 5-HT in the liver and small intestine.

### 3.4 SLBZS partially restores the perturbation of gut microbiota in NAFLD mice

**FIGURE 4:**
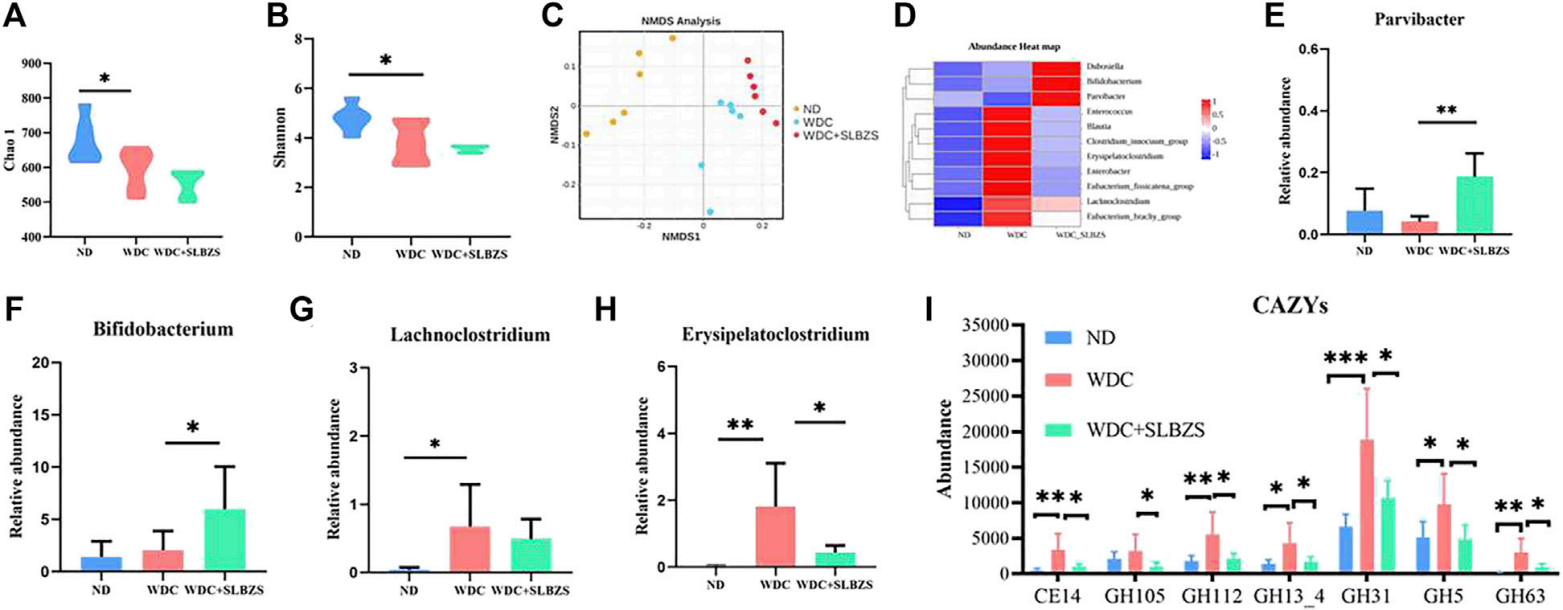
Regulation of SLBZS on gut microbiome in NAFLD mice. **(A‐B)** The violin plot of Chao1 and Shannon index. **(C)** Non-metric multidimensional scaling (NMDS) plots based on unweighted unifrac to compare β‐diversity of gut microbiota. Stress<0.1. **(D)** The abundance heat map at genus level of target differential gut microbiota in ND, WDC, WDC+SLBZS groups. **(E‐H)** The relative abundance of Parvibacter, Bifidobacterium, Lachnoclostridium, Erysipelatoclostridium. **(I)** The abundance of microbiota associated with seven carbohydrate enzyme families. The vertical axis represents relative abundance. Data are presented as the means ± SD. n=6 mice per group. *P <0.05, **P< 0.01, ***P < 0.001, ****P < 0.0001. P value obtained by one‐way ANOVA with Tukey’s post hoc tests.

To estimate the effects of SLBZS on the gut microbiota in NAFLD mice, the Chao1 and Shannon indexes were calculated to examine the α-diversity metrics. The differences between the control and NAFLD groups were significant for the Chao1 and Shannon indexes (Figures 4A, B). There was no significant difference in the α diversity between the NAFLD and SLBZS groups, indicating that SLBZS treatment did not enrich the diversity of gut microbiota.

Non-metric multidimensional scaling (NMDS) analysis based on the unweighted Unifrac index of β-diversity was performed to investigate the structural variations in the microbial communities across samples (Figure 4C). The results showed that there were distinct separations among the three groups, indicating that there may be different compositions of the gut microbiota among these three groups. To identify the target differential bacteria, the differential bacteria between the groups were evaluated. The final cladograms at the genus level are shown in Supplementary Figure S2C, which reveals different microbial communities in each group and a total of 47 identifiable differences. According to the LEfSe analysis, with LDA>2, *p* < 0.05, and species relative abundance >0.1%, there were a total of 11 genera with related changes in the trends (Figure 4D). Detailed information on all the changed genera is listed in Supplementary Table S4. As shown, *Bifidobacterium* and *Parvibacter*, which are helpful commensal microbiota, increased significantly with SLBZS intervention (Figures 4E, F). The harmful microbiota in NAFLD, including *Lachnoclostridium* and *Erysipelatoclostridium*, were elevated in NAFLD mice but were lowered by SLBZS treatment (Figures 4G, H). In addition, studies have shown that gut microbiota contain enzymes related to polysaccharide lysis (Ni et al., 2023). To investigate whether there were changes in the carbohydrate enzymes in this study, the common enzymes on the carbohydrate pathway were predicted; there were significant changes in the abundance of microbiota associated with seven carbohydrate enzyme families after SLBZS intervention (Figure 4I). The above results suggest that SLBZS promotes some beneficial bacteria while inhibiting some NAFLD-dependent taxa to improve hepatic steatosis. It is also suggested that carbohydrate enzymes may be involved in this process.

### 3.5 SLBZS partially restores the alteration of fecal metabolites in NAFLD mice

**FIGURE 5:**
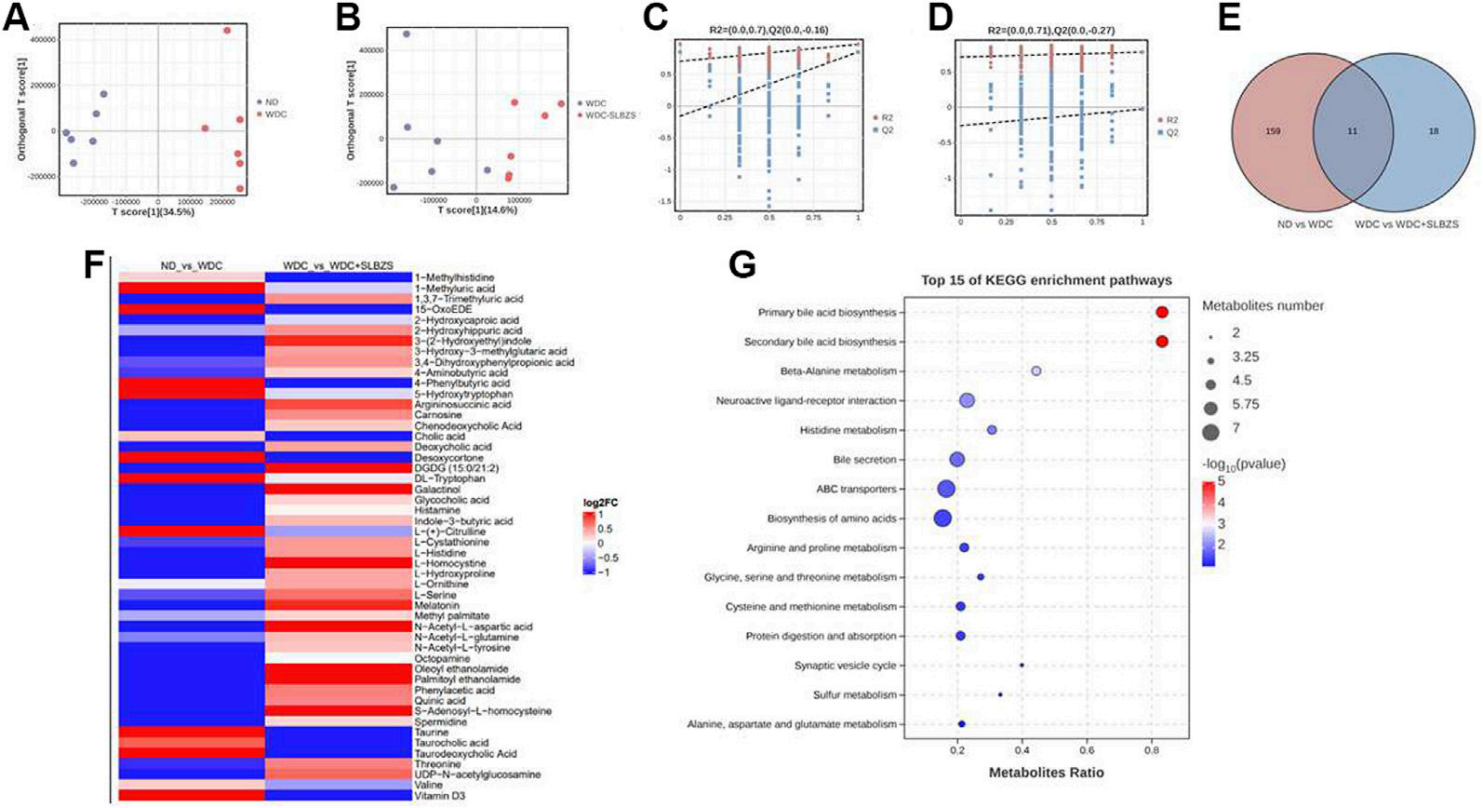
SLBZS altered the fecal metabolites in NAFLD mice. **(A, B)** OPLS‐DA score chart of pairwise comparisons. **(C, D)** Permutation test Chart of OPLS‐DA. **(E)** Venn diagram for common metabolites **(F)** The heatmap showed the 50 metabolites between two pairwise groups; the red color indicated up‐regulated metabolites and the blue color indicated down‐regulated metabolites. **(G)** Bubble pots of top 15 of KEGG pathway enrichment analysis of 50 metabolites.

Microbial metabolites have been proposed to be intermediate phenotypes that mediate interactions between the host and microbiome, thereby providing reliable and effective paths for gut microbiota activity. In this context, the OPLS-DA models revealed that there were distinct separations among all groups (Figures 5A, B), indicating that the profiles of the metabolites exhibit different patterns among the three groups. In addition, a permutation test suggested the good reliability of the OPLS-DA model for the control vs. NAFLD groups, with R2 (0.0, 0.7) and Q2 (0.0, −0.16). Moreover, the reliability results of the NAFLD vs. SLBZS groups were also ideal, with R2 (0.0, 0.71) and Q2 (0.0, −0.27) (Figures 5C, D).

A total of 2529 peaks were identified for further analyses. Based on the OPLS-DA models, metabolites with a variable importance value (VIP) > 1, *p*-value < 0.05, and fold change >1 or <1 were defined as significant differential metabolites among the three groups. Using a Venn diagram, 11 overlapped metabolites were obtained between two pairwise groups (Figure 5E). A total of 39 common metabolites were also screened for further analysis, including BAs, tryptophan metabolites, AAs, and fatty acids. There were a total of 50 metabolites that exhibited opposing patterns between the two pairwise comparisons (Supplementary Table S5). For example, compared with the control group, the NAFLD group showed upregulated levels of DL-tryptophan and 5-hydroxytryptophan, which were downregulated after SLBZS treatment. Conversely, the NAFLD group showed declined levels of 3-(2-hydroxyethyl) indole, melatonin, and indole-3-butyric acid that were upregulated after SLBZS treatment (Figure 5F). The alterations of fatty acids, polyamines, and purine derivatives in the NAFLD mice also tended to improve with SLBZS treatment.

These results indicate that SLBZS might reverse the alteration of metabolites in NAFLD mice. Before analyzing the correlations between the differential metabolites and gut microbiota, KEGG was applied, and there were 13 significant enrichment pathways (Figure 5G). These were primary BA biosynthesis, secondary BA biosynthesis, beta-alanine metabolism, neuroactive ligand–receptor interaction, histidine metabolism, bile secretion, ABC transporters, and biosynthesis of AAs; arginine and proline metabolisms; glycine, serine, and threonine metabolisms; cysteine and methionine metabolisms; protein digestion and absorption, and synaptic vesicle cycle.

### 3.6 NAFLD features are correlated with gut microbiota and metabolites restored by SLBZS

**FIGURE 6:**
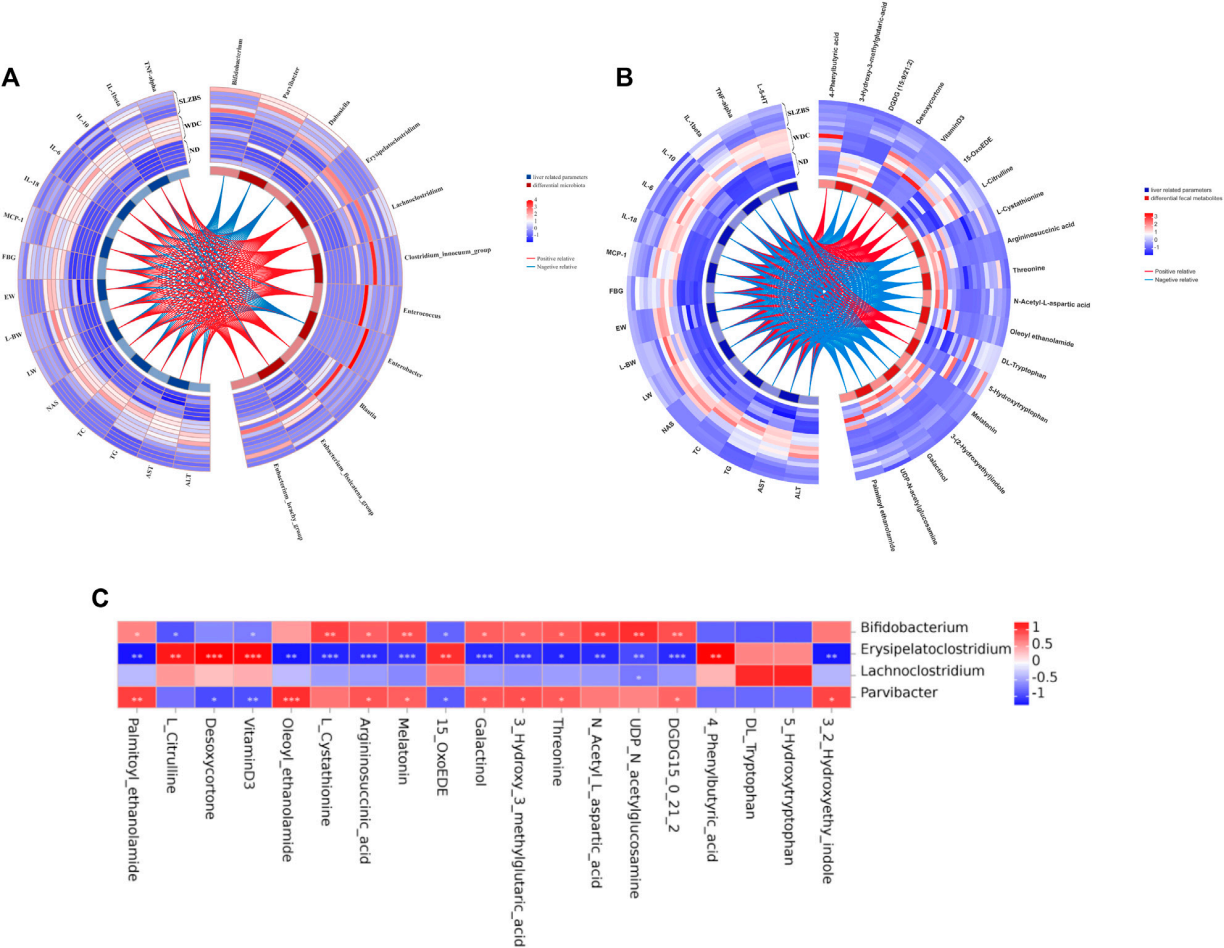
Spearman rank correlation analysis among SLBZS reversed gut microbiota, fecal metabolites and NAFLD features. **(A)** Heat-circos map of SLBZS reversed metabolites and liver-related parameters. **(B)** Heat-circos map of SLBZS reversed gut microbiota and liver-related parameters. **(C)** Correlation between 4 gut microbiota and 19 metabolites with significant change. *P <0.05, **P <0.01, ***P <0.001. AST: Serum aspartate transaminase. ALT: Serum alanine transaminase. TG: Liver triglyceride. FBG: Fast blood glucose. TC: Liver total cholesterol. NAS: NAFLD Activity Score. LW: Liver weight. L/W: Liver weight/Body weight (%). EW: Epididymal adipose weight.

All of the above mentioned results showed that SLBZS treatment could modulate fecal microbiota and metabolites, alleviating systemic inflammation and decreasing hepatic steatosis in NAFLD mice. To investigate the relationships among the SLBZS-restored gut microbiota, fecal metabolites, and NAFLD features, Spearman rank correlation analyses were performed. The results revealed that *Blautia*, *Erysipelatoclostridium*, and *Clostridium* innocuum group were positively correlated with NAFLD features, liver 5-HT, and inflammatory factors (Figure 6A), while *Bifidobacterium* and *Parvibacter* were negatively correlated. In addition, 4-phenylbutyric acid, vitamin D3, desoxycortone, and L-citrulline were positively correlated with liver function indexes, liver 5-HT, and inflammatory factors, while melatonin, 3-hydroxy-3-methylglutaric acid, and 3-(2-hydroxyethyl) indole were negatively correlated with them (Figure 6B). Gut microbiota like *Erysipelatoclostridium* and *Lachnoclostridium* were also positively correlated with 4-phenylbutyric acid, vitamin D3, desoxycortone, L-citrulline, DL-tryptophan, and 15-OxoEDE but negatively correlated with argininosuccinic acid and N-acetyl-L-glutamine (Figure 6C). These results indicated that SLBZS may improve NAFLD partially by modulating the gut microbiota and correlated metabolites.

## 4 Discussion

NAFLD is the most common chronic liver disease worldwide and affects more than a quarter of the global population as there are no FDA-approved therapies (Younossi et al., 2018). Studies have shown that SLBZS enhances insulin sensitivity in high-fat diet animals and inhibits the expression of TLR4, P38MAPK phosphorylation, and activation, thus exerting anti-inflammatory effects (Yang et al., 2014; Deng et al., 2019). However, the effects of SLBZS on NAFLD and its underlying mechanisms have remained unclear. The lack of clinically effective treatment plans and strategies for NAFLD highlights the urgency of exploring new drugs and promising treatment strategies to overcome existing problems (Zhou et al., 2020). In the present study, NAFLD and SLBZS-intervened models were constructed to evaluate the effects of SLBZS on NAFLD. Gut microbiota and metabolites in the fecal samples were also profiled to uncover the action mechanisms. The results show that SLBZS administration in mice with NAFLD markedly prevents hepatic steatosis and reduces inflammatory factors.

Moreover, emerging evidence shows that gut microbiota may be the therapeutic targets of TCM in treating NAFLD. In the present study, 11 gut genera that may be the therapeutic targets of SLBZS in treating NAFLD were identified. Dysbiosis in the gut microbiota caused by NAFLD was characterized by reduced alpha diversity and increase in potentially pathogenic microbes, including *Erysipelatoclostridium*, *Blautia*, *Lachnoclostridium*, and *Clostridium* innocuum group, which decreased after SLBZS administration. *Erysipelatoclostridium*, which is the core pro-inflammatory bacterial genus in NAFLD and significantly positive with liver 5-HT, has been reported to be positively correlated with HOMA-IR scores, inflammatory factors (Bailén et al., 2020; Ye et al., 2021; Hu et al., 2022), and liver fat changes in choline-deficient female subjects (Spencer et al., 2011). Moreover, *Erysipelatoclostridium* plays a critical role in elevating liver 5-HT level and the expression levels of genes moderating gut lipid storage (Bloemendaal et al., 2023), which may induce insulin resistance, steatosis, and oxidative stress through HTR2A (Oh et al., 2015; Choi et al., 2021; Li et al., 2023). In addition, *Lachnoclostridium* has been shown to be strongly and positively correlated with the progression of NAFLD, inflammatory factors, and adipocytokines (Cui et al., 2020; Yu et al., 2022; Jiao et al., 2023). In this study, the relative abundance of inflammation-related bacterial genus, such as *Erysipelatoclostridium* and *Lachnoclostridium*, was found to be higher in the NAFLD group and later decreased upon SLBZS treatment. The anti-inflammatory bacteria *Parvibacter* and anti-obesity bacteria *Bifidobacterium* increased significantly after SLBZS intervention. It was also found that *Bifidobacterium* was a beneficial probiotic for health (Shu et al., 2023) as it reduces insulin resistance, blood lipids, and obesity in HFD-induced obese mice (Safari and Gérard, 2019; Wang Y. et al., 2020; Liu D. et al., 2023) while balancing gut-derived 5-HT (Pirozzi et al., 2023) and negatively correlating with TC and low-density lipoprotein cholesterol (Zhang et al., 2021). A previous study reported that *Parvibacter* was a beneficial genus whose function could prevent obesity (Li Y. et al., 2022) and inhibit inflammatory factors (Pang et al., 2021). Interestingly, similar results were noted in this study. *Erysipelatoclostridium* was positively associated with inflammatory factors and liver 5-HT, but *Parvibacter* and *Bifidobacterium* were negatively associated with them. The above data indicate that *Parvibacter* and *Bifidobacterium* may play important roles in treating NAFLD.

NAFLD not only influences the host fecal microbiota but also regulates host metabolic homeostasis via microbial metabolites. Accordingly, several fecal metabolites including lipid metabolites, amino acids, and tryptophan metabolites were identified, which showed significant improvements after SLBZS intervention.

Lipid acquisition exceeding lipid removal can lead to liver steatosis, which involves an increase in fatty acid uptake and *de novo* lipid generation. However, the compensatory enhancement of fatty acid oxidation is not sufficient to normalize lipid levels, which could promote cell damage and disease progression through oxidative stress (Ipsen et al., 2018). It has been reported that vitamin D3, a steroid hormone mainly synthesized in the liver and involved in steroid biosynthesis, significantly increased in NAFLD mice, which is in agreement with the current results (Zhou et al., 2022). Studies have shown that oleoyl ethanol amide (OEA) constitutes a class of lipid compounds that also has anti-inflammatory and antioxidant functions (De Filippo et al., 2023; Tutunchi et al., 2023). Additionally, palmitoylethanolamide (PEA) is an endogenous lipid mediator that has been shown to reduce serum pro-inflammatory factors (Lama et al., 2021; Lama et al., 2022) while rebalancing 5-HT turnover through reshaping of the gut microbiota composition (Pirozzi et al., 2023). From the results of this study, it is noted that vitamin D3, desoxycortone, and 4-phenylbutyric acid were positively associated with *Erysipelatoclostridium* and *Lachnoclostridium*. PEA and OEA were negatively associated with pro-inflammatory bacteria, liver 5-HT, and liver parameters, while being significantly positively associated with *Parvibacter*.

It was also found that a batch of AAs (including L-citrulline and DL-tryptophan) and their correlated gut microbiota were altered in NAFLD and ameliorated by SLBZS. The KEGG pathway analysis indicated that 14 altered metabolites were mainly enriched in the AA metabolic pathways. Furthermore, it has been reported that taurine, histamine, and spermine could increase inflammatory factors such as IL-8 (Wu et al., 2021) and branched-chain AAs such as valine, which are related to insulin resistance (Masoodi et al., 2021). Moreover, serine as an upstream substance of tryptophan was significantly downregulated, causing tryptophan upregulation in NASH (Lai et al., 2015; Dorochow et al., 2023). Another amnio metabolic pathway, namely, tryptophan metabolism that has been found to be a critical pathway for metabolism in hepatic inflammatory infiltration, was related to the course of NAFLD (Lai et al., 2015; Hu et al., 2021). This encompasses serotonin metabolism, whose downstream metabolite melatonin has protective effects against fibrosis and inflammation (Liu et al., 2021). In the results of this study, tryptophan was elevated and positively associated with inflammatory factors and *Lachnoclostridium*, while indole-3-butyric acid and melatonin were reduced in NAFLD mice and restored by SLBZS. It is therefore suggested that SLBZS treatment affects gut microbiota-mediated AA metabolism, which might be the potential target of NAFLD.

This study is also noted to have several limitations: 1) Although the intervention dose of SLZBS used in this study was within the clinically effective range, there is still no uniform standard for a safe dose of SLZBS. 2) Specific species of bacteria or fecal metabolites still need to be verified further for NAFLD mice. To elucidate the causal relationships between the fecal metabolites and gut microbiota in the NAFLD models, further in-depth studies are needed.

## 5 Conclusion

Generally, the preliminary findings of this study are that SLBZS remarkably increases the relative abundance of probiotics (*Bifidobacterium* and *Parvibacter*) and inhibits the growth of pro-inflammatory bacteria (*Erysipelatoclostridium* and *Lachnoclostridium*) in NAFLD mice. Meanwhile, lipid metabolites such as 15-OxEDE, vitamin D3, desoxycortone, and OEA were restored by SLBZS. Thus, SLBZS might improve NAFLD by modulating gut microbiota, gut-derived 5-HT, and their correlated metabolites to decrease fat accumulation in the liver and inflammatory responses.

## Data Availability

The datasets presented in this study can be found in online repositories. The names of the repository/repositories and accession number(s) can be found at: NCBI with BioProject ID PRJNA1100708 and Supplementary Material.
