# Supplementary material for: Shenling Baizhu San ameliorates non-alcoholic fatty liver disease in mice by modulating gut microbiota and metabolites
Source: Front Pharmacol. 2024 Apr 24;15:1343755. doi: 10.3389/fphar.2024.1343755 (PMC11076757; doi:10.3389/fphar.2024.1343755)
Supplement: Supplementary file 1 [file DataSheet1.pdf]

frontiers

Supplementary Material

1 Supplementary Figures and Tables

1.1 Supplementary Figures

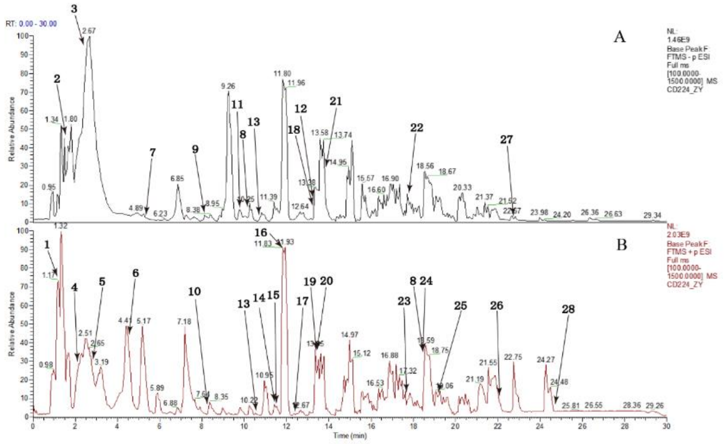

Supplementary Figure 1. Total ion chromatogram of SLZBS obtained by UHPLC-MS/MS analysis in (A) positive ion mode and (B) negative ion mode.

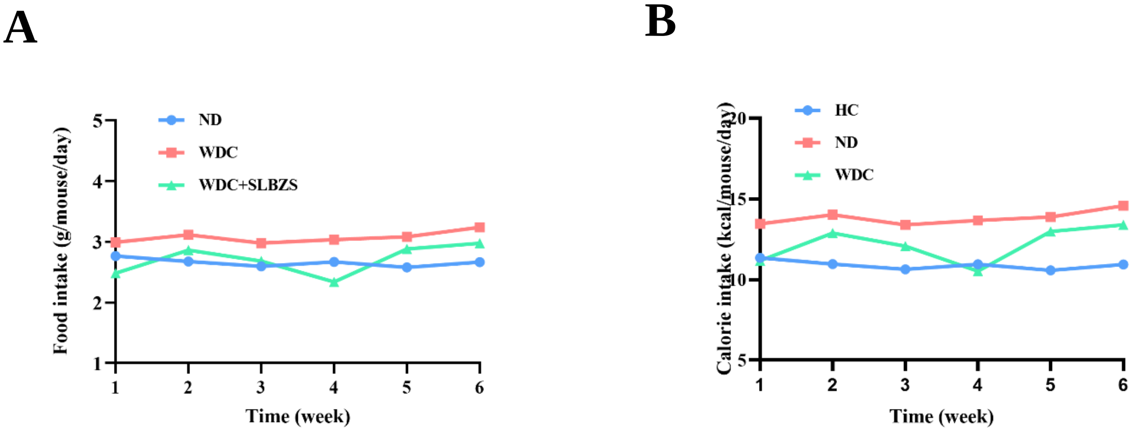

C

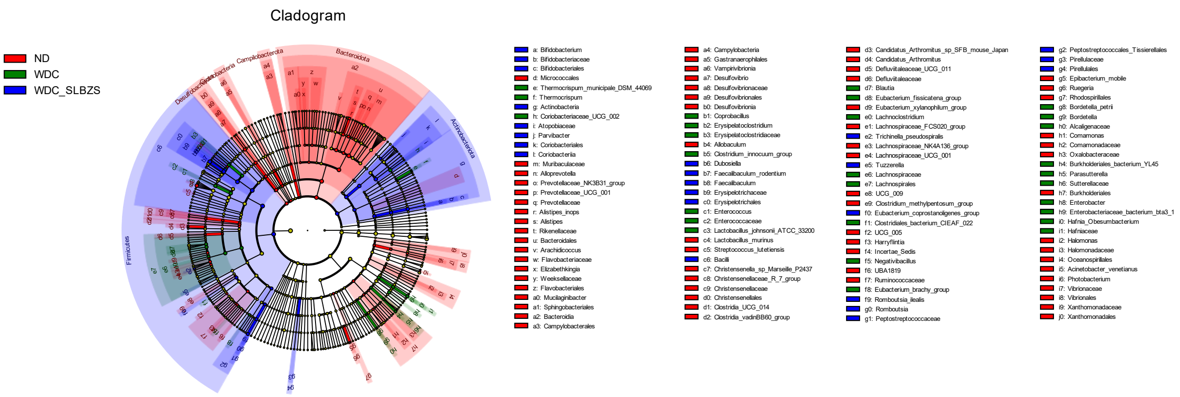

Supplementary Figure 2. Food intake and energy intake of each group in 6 weeks. (A) Food intake(g/mouse/day). (B) Calorie intake(kcal/mouse/day). (C) Plot cladogram.

1.2 Supplementary Tables

Supplementary Tables 1. The composition of SLBZS.

Chinese name	English name	Latin name of plants	Medical part	Proportion
Ren Shen	Ginseng	Panax ginseng C.A.Mey.	Root	5
Fu Ling	Poria	Poria cocos (Schw.) Wolf	Sclerotium	5
Bai Zhu	Atractylodes Rhizome	Atractylodes macrocephala Koidz.	Rhizome	5
Shan Yao	Dioscorea Rhizome	Dioscorea opposita Thunb.	Rhizome	5
Bai Bian Dou	Dolichos Seed	Dolichos lablab L.	Seed	4
Lian Zi	Nelumbo Seed	Nelumbo nucifera Gaertn.	Seed	3
Yi Yi Ren	Yi Yi Ren	Coix lacryma-jobi L. var. ma-yuen. (Rom.Caill.) Stapf	Kernel	3
Sha Ren	Amomum Fruit	Amomum villosum Lour.	Fruit	2
Jie Geng	Jie Geng	Platycodon grandiflorus (Jacq.) A. DC.	Root	2
Zhi Gan Cao	Glycyrrhiza	Glycyrrhiza uralensis Fisch.	Root and rhizome	3

Supplementary Tables 2. The gradient elution condition

Time/min	A%	B%
1	98	2
5	80	20
10	50	50
15	20	80
20	5	95
27	5	95
28	98	2
30	98	2

Supplementary Tables 3. Identification of chemical constituents from SLZBS by UHPLC-MS/MS

NO.	Name	Molecular formula	RT (min)	Mass error (ppm)	Ionization model
1	Choline	C5H13N O	1.217	3.7	[M+H]+1
2	D-(-)-Quinic acid	C7H12O6	1.55	-5.2	[M-H]-1
3	Citric acid	C6H8O7	2.641	＜0.1	[M-H]-1
4	Nicotinic acid	C6H5NO2	2.149	1.4	[M+H]+1
5	4-Hydroxybenzaldehyde	C7H6O2	2.791	1.3	[M+H]+1
6	Adenosine	C10H13N5O4	4.432	-1.5	[M+H]+1
7	Gallic acid	C7H6O5	5.148	-5.8	[M-H]-1
8	Caffeic acid	C9H8O4	10.15/18.542	-4.8	[M-H]-1/ [M+H]+1
9	Folinic acid	C20H23N7O7	8.116	-8.9	[M-H]-1
10	Codeine	C18H21NO3	8.301	＜0.1	[M+H]+1
11	Benzoic acid	C7H6O2	9.684	-11.0	[M-H]-1
12	Naringin	C27H32O14	12.902	-0.1	[M-H]-1
13	Vanillin	C8H8O3	10.569/10.545	1.5	[M-H]-1/

					[M+H]+1
14	Coumarin	C9H6O2	11.431	-0.4	[M+H]+1
15	7-hydroxy-6-methoxy-2H-chromen-2-one	C10H8O4	11.438	＜0.1	[M+H]+1
16	Isoliquiritigenin	C15H12O4	11.907	-3.0	[M+H]+1
17	Safrole	C10H10O2	12.382	1.2	[M+H]+1
18	Rutin	C27H30O16	12.938	-0.7	[M-H]-1
19	Formononetin	C16H12O4	13.366	-18.8	[M+H]+1
21	Luteolin	C15H10O6	13.967	-9.9	[M-H]-1
22	Ginsenoside Rb1	C54H92O23	17.721	-1.0	[M-2H]-2
23	Licochalcone A	C21H22O4	17.789	-1.5	[M+H]+1
24	Dibutyl phthalate（DBP）	C16H22O4	18.54	-1.1	[M+H]+1
25	Ursolic acid	C30H48O3	19.161	-0.8	[M+H]+1
26	Monoolein	C21 H40 O4	22.173	-1.1	[M+Na]+1
27	Ostruthin	C19H22O3	22.641	10.6·	[M-H]-1
28	Ergosterol peroxide	C28H44O3	24.687	-0.8	[M+H]+1

Supplementary Tables 4. Significantly changed gut microbiota among three groups

**Genus name**	**Relative abundance**			***P*-value**	**LAD score**
	**Control**	**NAFLD**	**WDC+SLBZS**		
Dubosiella	5.299	6.607	12.878	0.042	4.624
Bifidobacterium	1.417	2.051	5.994	0.038	4.332

Enterococcus	0.166	6.098	1.753	0.008	4.506
Enterobacter	0.326	5.798	1.007	0.035	4.549
Eubacterium_fissicatena_group	0.776	2.842	1.026	0.045	4.033
Blautia	0.117	2.080	0.616	0.002	3.999
Erysipelatoclostridium	0.040	1.816	0.437	0.001	3.926
Lachnoclostridium	0.042	0.676	0.490	0.005	3.545
Clostridium_innocuum_group	0.005	0.510	0.135	0.003	3.475
Eubacterium_brachy_group	0.006	0.105	0.057	0.028	2.757
Parvibacter	0.077	0.042	0.186	0.011	2.925

Supplementary Tables 5. 50 overlapped changed metabolites between pairwise groups

**Super Class**	**Metabolite**	**log_2FC(NAFLD/ND)**	**#*P***	**log_2FC(SLBZS/NAFLD)**	**##*P***
Benzenoids	Octopamine	-2.364	0.000	0.007	0.970
Benzenoids	4-Phenylbutyric acid	2.047	0.035	-1.482	0.069
Benzenoids	2-Hydroxyhippuric acid	-0.435	0.286	0.539	0.227
Benzenoids	Phenylacetic acid	-3.996	0.002	0.614	0.135
Lipids and lipid-like molecules	Taurocholic acid	0.769	0.282	-1.029	0.136
Lipids and lipid-like molecules	***3-Hydroxy-3-methylglutaric acid**	-2.389	0.000	0.469	0.042
Lipids and lipid-like molecules	Chenodeoxycholic Acid	-1.390	0.006	0.246	0.576
Lipids and lipid-like molecules	Cholic acid	0.298	0.731	-1.512	0.174

Lipids and lipid-like molecules	2-Hydroxycaproic acid	-2.018	0.009	-0.192	0.704
Lipids and lipid-like molecules	Deoxycholic acid	-2.241	0.000	0.450	0.324
Lipids and lipid-like molecules	***DGDG (15:0/21:2)**	-4.443	0.000	2.462	0.044
Lipids and lipid-like molecules	***Desoxycortone**	2.503	0.005	-1.250	0.033
Lipids and lipid-like molecules	***Vitamin D3**	4.961	0.039	-3.586	0.049
Lipids and lipid-like molecules	Methyl palmitate	-0.434	0.029	0.216	0.237
Lipids and lipid-like molecules	Taurodeoxycholic Acid	3.995	0.278	-4.102	0.275
Lipids and lipid-like molecules	Glycocholic acid	-2.446	0.022	0.207	0.756
Lipids and lipid-like molecules	***15-OxoEDE**	2.206	0.001	-1.105	0.004
Organic acids and derivatives	N-Acetyl-L-tyrosine	-3.751	0.000	0.273	0.423
Organic acids and derivatives	1-Methylhistidine	0.215	0.772	-2.196	0.121
Organic acids and derivatives	N-Acetyl-L-glutamine	-0.646	0.026	0.331	0.234
Organic acids and derivatives	***Palmitoyl ethanolamide**	-2.636	0.020	1.677	0.010
Organic acids and derivatives	***L- (+)-Citrulline**	1.453	0.000	-0.508	0.037
Organic acids and derivatives	***L-Cystathionine**	-0.885	0.002	0.485	0.016

Organic acids and derivatives	Carnosine	-1.148	0.125	0.556	0.404
Organic acids and derivatives	***Argininosuccinic acid**	-3.833	0.001	0.847	0.023
Organic acids and derivatives	L-Histidine	-1.555	0.014	0.488	0.381
Organic acids and derivatives	Valine	0.246	0.532	-0.499	0.170
Organic acids and derivatives	L-Hydroxyproline	-2.066	0.012	0.434	0.170
Organic acids and derivatives	***Threonine**	-0.955	0.018	0.608	0.037
Organic acids and derivatives	L-Homocystine	-4.695	0.007	1.350	0.057
Organic acids and derivatives	***N-Acetyl-L-aspartic acid**	-3.688	0.016	2.308	0.028
Organic acids and derivatives	Taurine	1.395	0.084	-1.648	0.052
Organic acids and derivatives	L-Ornithine	-0.089	0.813	0.432	0.180
Organic acids and derivatives	4-Aminobutyric acid	-0.910	0.141	0.204	0.552
Organic acids and derivatives	L-Serine	-0.829	0.087	0.683	0.069
Organic nitrogen compounds	***Oleoyl ethanolamide**	-2.821	0.034	1.279	0.006
Organic nitrogen compounds	Histamine	-2.869	0.010	0.048	0.863
Organic nitrogen compounds	Spermidine	-3.007	0.001	0.194	0.595

Organoheterocyclic compounds	DL-Tryptophan	1.303	0.045	-0.095	0.786
Organoheterocyclic compounds	5-Hydroxytryptophan	2.144	0.079	-0.197	0.813
Organoheterocyclic compounds	***Melatonin**	-3.198	0.001	0.952	0.000
Organoheterocyclic compounds	3-(2-Hydroxyethyl) indole	-3.533	0.000	0.959	0.193
Organoheterocyclic compounds	1-Methyluric acid	2.591	0.007	-0.220	0.592
Organoheterocyclic compounds	1,3,7-Trimethyluric acid	-2.729	0.001	0.538	0.195
Organoheterocyclic compounds	S-Adenosyl-L-homocysteine	-1.405	0.001	2.163	0.074
Organoheterocyclic compounds	Quinic acid	-2.736	0.001	0.595	0.122
Organoheterocyclic compounds	Indole-3-butyric acid	-1.331	0.003	0.331	0.359
Organic oxygen compounds	***Galactinol**	-3.085	0.002	1.108	0.007
Nucleosides, nucleotides, and analogues	***UDP-N-acetylglucosamine**	-1.721	0.006	0.731	0.035
Phenylpropanoids and polyketides	3,4-Dihydroxyphenylpropionic acid	-0.826	0.071	0.520	0.096

#*P*： the *P* value of NAFLD-vs.-ND group; ##*P*: the *P* value of SLBZS-vs.-NAFLD group.
*: overlapped significantly changed metabolites between pairwise groups.

Supplementary Table 6. main reagents and kits used in this study

Reagent	Resource	Identifier
Carbon tetrachloride (CCL_4)	Sinopharm Chemical Reagent Co., Ltd., China	Cat#: 10006464
Corn oil	Sigma	Cat#: 8001-30-7
Ethyl Alcohol	FuYu Chemical,China	Cat#: 64-17-
Dimethylbenzene	Guangzhou Chemical reagent factory,China	Cat#:1330-20-7
Haematoxylin staining solution	Biosharp,China	Cat#: BL702A
Eosin Y Solution	Solarbio,China	Cat#: G1100
Rhamsan gum	Sinopharm Chemical Reagent Co., Ltd., China	Cat#: 10004160
Glycerol Jelly Mounting Medium	Beyotime,China	Cat#: C0187
Primary antibody: E-cad	Wuhan Servicebio Technology CO., LTD, China	Cat#: GB12083
Secondary antibody: HRP-labeled goat anti-rabbit IgG	Wuhan Servicebio Technology CO., LTD, China	Cat#: GB23303
**Critical commercial assays**	**Resource**	**Identifier**
Serum alanine aminotransferase (ALT)	Nanjing Jian Cheng Bioengineering Institute, China	Cat#: C009-2-1
Serum aspartate aminotransferase (AST)	Nanjing Jian Cheng Bioengineering Institute, China	Cat#: C010-2-1
Hepatic triglyceride (TG)	Nanjing Jian Cheng Bioengineering Institute, China	Cat#: A110-1-1

Hepatic total cholesterol (TC)	Nanjing Jian Cheng Bioengineering Institute, China	Cat#: A111-1-1
RNA extraction kit	Vazyme, America	Cat#: RC101
DNA conversion kit	Vazyme, America	Cat#: R223-01
Quantitative PCR kit	Vazyme, America	Cat#: Q712
Glucose kit	Accu-Chek,Hong Kong	Cat#: L0140
Oil Red O Stain Kit	Nanjing Jian Cheng Bioengineering Institute, China	Cat#: D027-1-1
Histochemistry kit DAB colorant	Wuhan Servicebio Technology CO., LTD, China	Cat#: G1212
